# *Lavandula stoechas* Ethanol Extracts Induce Apoptosis in Breast, Bladder, and Glioblastoma Cancer Cells

**DOI:** 10.3390/pharmaceutics18040500

**Published:** 2026-04-18

**Authors:** Ihsan Nalkiran, Hatice Sevim Nalkiran

**Affiliations:** Department of Medical Biology, Faculty of Medicine, Recep Tayyip Erdogan University, Rize 53020, Türkiye; ihsan.nalkiran@erdogan.edu.tr

**Keywords:** *Lavandula stoechas*, natural products, apoptosis, clonogenic survival, triple-negative breast cancer, bladder cancer, glioblastoma

## Abstract

**Background:** *Lavandula stoechas* has attracted increasing attention for its potential anticancer properties; however, evidence regarding its effects on apoptotic signaling across different tumor types remains limited. **Methods:** In this study, the effects of dry and fresh ethanol extracts of *Lavandula stoechas* L. subsp. stoechas (LsDE and LsFE) were investigated in MDA-MB-231 triple-negative breast cancer, RT4 bladder carcinoma, and T98G glioblastoma cell lines, providing a comparative evaluation of their apoptotic effects. Long-term proliferative capacity was assessed using clonogenic survival assays, while apoptosis-related responses were evaluated by Annexin V–FITC/propidium iodide staining, quantitative RT-PCR of *BAX* and *BCL2* and Western blot analysis of Bax, Bcl-2, and cleaved PARP1. **Results:** Both extracts significantly reduced clonogenic survival in all tested cancer cell lines, with LsDE showing stronger inhibitory effects in RT4 and T98G cells. Annexin V/PI analysis revealed cell type-dependent response patterns. In MDA-MB-231 cells, both extracts increased the proportion of PI-positive cells, suggesting a loss of membrane integrity, whereas RT4 cells exhibited increased early apoptotic and membrane-compromised populations. In contrast, T98G cells showed comparatively limited changes associated with apoptosis. Transcriptional analysis demonstrated extract- and cell line-specific modulation of the *BAX/BCL2* ratio. Western blot analysis further demonstrated activation of mitochondrial apoptotic signaling through coordinated regulation of Bax and Bcl-2 and increased PARP1 cleavage. LsFE showed the strongest apoptosis-associated changes in MDA-MB-231 cells, whereas LsDE showed stronger effects in T98G cells, while both extracts were effective in modulating these proteins in RT4 cells. **Conclusions:** These findings indicate that ethanol extracts of *L. stoechas* impair long-term proliferative capacity and induce tumor type-dependent modulation of apoptosis-related markers. This study provides an integrated experimental framework that combines clonogenic survival assays, apoptosis analyses, gene expression, and protein-level measurements, supporting further investigation of *L. stoechas* extracts in cancer research.

## 1. Introduction

Cancer is a multifactorial disease characterized by uncontrolled cell proliferation, sustained survival signaling, and the ability to evade programmed cell death [1]. One hallmark of malignant transformation is resistance to apoptosis, which allows genetically unstable cells to persist and accumulate further oncogenic alterations [2,3]. Dysregulation of intrinsic apoptotic pathways, particularly an imbalance between pro-apoptotic and anti-apoptotic BCL-2 family proteins, contributes to tumor progression and therapeutic resistance in multiple cancer types [4,5]. Therefore, restoration of apoptotic signaling has become a central strategy in the development of anticancer therapies. Although substantial progress has been achieved with chemotherapy, targeted therapy, and immunotherapy, clinical outcomes are frequently limited by drug resistance and tumor heterogeneity [6,7]. These challenges reinforce the need for new therapeutic approaches that can effectively modulate cancer cell survival pathways.

Natural products have historically played a critical role in anticancer drug discovery. A significant proportion of clinically used anticancer agents originate from natural sources or are structurally inspired by plant-derived compounds [8,9]. Secondary metabolites such as flavonoids, terpenoids, alkaloids, and phenolic acids have been shown to regulate oxidative stress, interfere with cell cycle progression, and induce apoptosis through intrinsic and extrinsic pathways [10,11]. The structural diversity of phytochemicals provides a broad chemical landscape for identifying bioactive molecules that selectively target tumor cell-specific molecular pathways [12]. Plant extracts have been extensively reported in the literature to contain bioactive phytochemicals with anticancer potential across various malignancies, particularly glioblastoma, bladder cancer, and breast cancer [13,14,15,16,17].

Among medicinal plant families, Lamiaceae family has attracted attention for its rich phytochemical content and broad spectrum of biological activities. *Lavandula stoechas* (*L. stoechas*), an aromatic plant native to the Mediterranean region, contains terpenoids, flavonoids, and phenolic derivatives and has been reported to exhibit antioxidant, anti-inflammatory, antimicrobial, and cytotoxic properties [14,18,19]. Our recent work demonstrated the cytotoxic effect of ethanol extracts of *Lavandula stoechas* L. subsp. stoechas (*L. stoechas* L.), including a dry ethanol extract (LsDE) and a fresh ethanol extract (LsFE), and characterized their phytochemical composition [14]. However, the molecular mechanisms underlying extract-induced growth suppression and apoptosis remain unclear. To address this gap, the present study aimed to characterize the mechanisms underlying the effects of LsFE and LsDE through integrated analysis of clonogenic survival, Annexin V/PI-based apoptosis, regulation of apoptosis-related genes (*BAX* and *BCL2*), and protein-level validation including PARP cleavage.

## 2. Materials and Methods

### 2.1. Lavandula stoechas Extracts

*Lavandula stoechas* L. subsp. stoechas (*L. stoechas* L.) plant material was collected in Muğla, Türkiye, and taxonomically identified as previously described [14]. Detailed information regarding plant authentication, extraction procedures, and phytochemical characterization has been reported in this previous study. The ethanol extracts used in the present study were derived from the same batch characterized by LC–MS analysis. To ensure consistency, extracts were prepared in bulk, aliquoted, and stored at −20 °C in DMSO until use, minimizing batch-to-batch variability [14]. Briefly, aerial flowering parts of the plant were extracted using ethanol, followed by filtration and lyophilization to obtain dried extract powders. In the present study, the dry ethanol extract (LsDE) and fresh ethanol extract (LsFE) were used for biological assays. Lyophilized extracts were dissolved in DMSO to prepare stock solutions (50 mg/mL) and subsequently diluted in culture medium to obtain the desired working concentrations. In the present study, the LsDE was prepared from shade-dried plant material, whereas the LsFE was obtained from freshly collected plant material without a drying step. Both extracts were analyzed to determine whether differences in extract preparation influence biological responses.

### 2.2. Cell Culture Conditions

The human cancer cell lines used in this study included MDA-MB-231 (breast adenocarcinoma), RT4 (bladder carcinoma), and T98G (glioblastoma). T98G cells were obtained from the American Type Culture Collection (ATCC, Manassas, VA, USA), whereas MDA-MB-231 and RT4 cells were provided by collaborating laboratories. Cells were cultured at 37 °C in a humidified atmosphere containing 5% CO_2_. Each cell line was maintained in its appropriate growth medium supplemented with 10% fetal bovine serum (FBS; Sigma-Aldrich, St. Louis, MO, USA) and 1% penicillin–streptomycin. Specifically, MDA-MB-231 and RT4 cells were grown in RPMI-1640 medium, while T98G cells were cultured in high-glucose Dulbecco’s Modified Eagle Medium (DMEM; Gibco, Thermo Fisher Scientific, Inc., Waltham, MA, USA).

### 2.3. Clonogenic Survival Assay

MDA-MB-231, RT4, and T98G cells were seeded in 12-well plates at a density of 250 cells per well in complete growth medium supplemented with 10% FBS. Cells were allowed to adhere overnight under standard culture conditions (37 °C, 5% CO_2_). Following attachment, cells were treated with the IC50 values (µg/mL) of *L. stoechas* L. extracts determined at 48 h post-treatment (for LsDE 1.40 µg/mL for RT4, 0.63 µg/mL for MDA-MB-231 and 0.58 µg/mL for T98G and for LsFE for RT4 1.72 µg/mL, for MDA-MB-231 0.28 µg/mL and for T98G 0.43 µg/mL) [14]. IC50 values used for dose selection were derived from our previous study performed under comparable experimental conditions, using the same cell lines within similar passage ranges and the same extract batch. These values were used to guide the selection of biologically relevant concentrations for mechanistic analyses rather than being re-determined in the present study. DMSO was used as the corresponding vehicle control at the same final concentration as in extract-treated groups. After treatment, cells were incubated for 7–10 days to allow colony formation. At the end of the incubation period, colonies were gently washed with phosphate-buffered saline (PBS; Gibco; Thermo Fisher Scientific), fixed with methanol for 15 min at room temperature, and stained with Giemsa solution (Giemsa’s azur–eosin–methylene blue solution, #109203, Sigma-Aldrich, St. Louis, MO, USA). Excess stain was removed by washing with distilled water, and plates were air-dried. Colonies consisting of more than 50 cells were counted manually using the Cell Counter plugin in ImageJ software (v1.54g; National Institutes of Health, Bethesda, MD, USA). Clonogenic survival was expressed relative to the corresponding DMSO control.

### 2.4. Annexin V/PI Staining

MDA-MB-231, RT4, and T98G cells were seeded onto sterile round coverslips placed in 12-well plates and allowed to adhere for 16 h. Cells were then treated with IC50 doses of LsDE and LsFE for 48 h, with DMSO used as the corresponding vehicle control. Following treatment, cells were stained with Annexin V–FITC and propidium iodide (PI) using an Annexin V–FITC Apoptosis Detection Kit (Abcam, Cambridge, UK, cat. no. ab14085) according to the manufacturer’s instructions and visualized. Images were captured from randomly selected fields for each experimental group using appropriate FITC and PI filter settings. Separate fluorescence channels were acquired for Annexin V–FITC (green) and PI (red), and channel merging was performed using ImageJ software (NIH, USA) to generate composite images. Quantitative analysis was conducted using ImageJ. Cells were manually counted using the Cell Counter plugin and classified according to fluorescence signals as early apoptotic (Annexin V^+^/PI^−^), late apoptotic (Annexin V^+^/PI^+^), or membrane-compromised/necrotic (Annexin V^−^/PI^+^). The percentage of cells in each category was calculated relative to the total number of cells counted per field on brightfield images, and mean values were obtained from independent experiments.

### 2.5. Quantitative Real-Time Polymerase Chain Reaction (qRT-PCR)

Total RNA was isolated using a commercial RNA isolation kit (Macherey-Nagel, Düren, Germany) according to the manufacturer’s instructions. RNA concentration and purity were determined using µDrop plates on a Thermo Multiskan Go spectrophotometer (Thermo Fisher Scientific, Waltham, MA, USA). Complementary DNA (cDNA) was synthesized using the High-Capacity cDNA Reverse Transcription Kit (Applied Biosystems, Carlsbad, CA, USA), following the manufacturer’s protocol. Quantitative real-time PCR (qRT-PCR) was performed in 96-well optical plates using LightCycler 480 Probes Master (Roche Diagnostics, Mannheim, Germany) on a LightCycler 480 II system (Roche Diagnostics). Detailed reaction conditions were described in our previous study [20]. TaqMan probe-based assays for *BAX* (*BCL2L4*, cat. no. 142318) and *BCL2* (cat. no. 100083) were purchased from Roche (Mannheim, Germany). GAPDH was used as the reference gene for normalization. *GAPDH* primer and probe sequences were as follows: forward primer (5′-GAAGGTGAAGGTCGGAGTC-3′), reverse primer (5′-GAAGATGGTGATGGGATTTC-3′), and YAK-labeled probe (YAK-CAAGCTTCCCGTTCTCAGCCT-BBQ) (TIB MOLBIOL, Berlin, Germany). Relative gene expression levels were calculated using the 2^−ΔΔCt^ method.

### 2.6. Western Blot Analysis

Cells were treated with LsDE, LsFE, or DMSO control and lysed in RIPA buffer supplemented with a protease inhibitor cocktail on ice for 20–30 min. Lysates were cleared by centrifugation at 13,000× *g* for 10–15 min at 4 °C, and protein concentrations were determined using the Pierce Rapid Gold BCA Protein Assay Kit (Thermo Fisher Scientific, Cat. No. A55861). Equal amounts of protein (20–30 µg) were separated on 10–12% SDS–PAGE gels and transferred to PVDF membranes. Membranes were blocked with 5% non-fat milk in TBST for 1 h at room temperature and incubated overnight at 4 °C with the following primary antibodies: anti-Bax (Invitrogen, Carlsbad, CA, USA; Cat. No. MA5-32031; 1:1000), BCL2 Monoclonal Antibody (Elabscience, Houston, TX, USA; Cat. No. E-AB-22004; 1:1000), anti-cleaved PARP1 (Asp214/215) (Thermo Fisher Scientific, Waltham, MA, USA; Cat. No. 44-698G; 1:1000), and anti-β-tubulin (Cell Signaling Technology, Danvers, MA, USA; Cat. No. 2146S; 1:1000). After washing, membranes were incubated with HRP-conjugated secondary antibodies, including HRP-conjugated anti-mouse IgG (Cell Signaling Technology, Danvers, MA, USA; Cat. No. 7076S) and Goat Anti-Rabbit IgG H&L (HRP) (Abcam, Cambridge, UK; Cat. No. ab205718), for 1 h at room temperature. Protein bands were developed using Clarity Western ECL Substrate (Bio-Rad Laboratories, Hercules, CA, USA; Cat. No. 1705060) and visualized with the ChemiDoc Imaging System (Bio-Rad Laboratories, Hercules, CA, USA). Band intensities were quantified using ImageJ software (National Institutes of Health, Bethesda, MD, USA) and normalized to β-tubulin. All experiments were performed in triplicate.

### 2.7. Statistical Analysis

Statistical analyses for clonogenic survival assays, Annexin V/PI apoptosis assays, and qRT-PCR experiments were performed using SPSS software (version 25.0; IBM Corp., Armonk, NY, USA). Differences among groups were evaluated by one-way analysis of variance (ANOVA) followed by Tukey’s post hoc test for comparisons between extract-treated groups and their corresponding DMSO controls. Statistics of Western blot densitometric analyses were performed using GraphPad Prism software (version 8.0.1, GraphPad Software, San Diego, CA, USA) using one-way ANOVA followed by Tukey’s post hoc test. Data are presented as mean ± standard deviation (SD). All experiments were conducted in at least three independent replicates. A *p*-value < 0.05 was considered statistically significant.

## 3. Results

### 3.1. L. stoechas L. Extracts Reduce Clonogenic Survival with Extract- and Cell Line-Specific Potency

**Figure 1:**
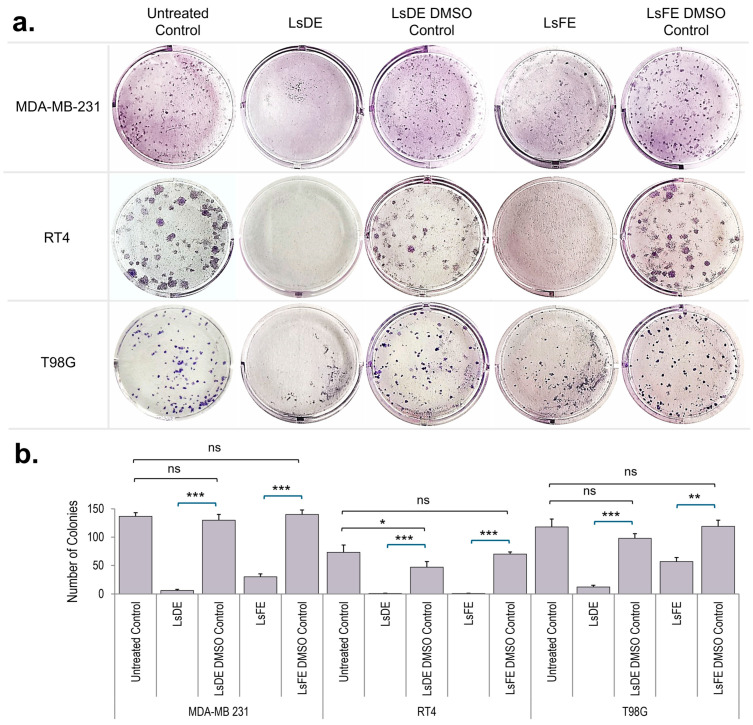
Effects of LsDE and LsFE on the clonogenic survival of MDA-MB-231, RT4, and T98G cancer cells. (**a**) Representative colony formation assay images showing the clonogenic potential of cancer cells following treatment with LsDE, LsFE, and their respective DMSO vehicle controls, compared with untreated control groups. Colonies were visualized by crystal violet staining. (**b**) Quantification of colony numbers in each treatment group. Data are presented as mean ± SEM from three independent experiments. Statistical comparisons were performed between each extract and its corresponding DMSO vehicle control using one-way ANOVA followed by Tukey’s post hoc test. ns, not significant; * *p* < 0.05; ** *p* < 0.01; and *** *p* < 0.001.

The effects of LsDE and LsFE on long-term clonogenic survival were evaluated in MDA-MB-231, RT4, and T98G cancer cell lines (Figure 1a,b). In MDA-MB-231 cells, LsDE treatment significantly reduced colony formation compared with its DMSO control (*p* < 0.001), indicating strong inhibition of clonogenic survival. LsFE also significantly decreased colony formation relative to its corresponding DMSO control (*p* < 0.001), although more residual colonies remained than in the LsDE group. No significant differences were observed between untreated and DMSO control groups, confirming that the inhibitory effects resulted from the extracts rather than the solvent. In RT4 cells, LsDE treatment nearly completely abolished colony formation and showed a highly significant reduction compared to its vehicle control (*p* < 0.001), demonstrating strong suppression of clonogenic survival. LsFE also significantly decreased colony numbers relative to its DMSO control (*p* < 0.05), although the inhibitory effect was weaker than that observed with LsDE. No significant differences were observed between untreated and vehicle control groups. In T98G cells, both extracts significantly decreased colony formation compared to their respective DMSO controls. LsDE caused a strong reduction (*p* < 0.001), while LsFE also significantly suppressed colony formation (*p* < 0.01) but to a lesser degree than LsDE. Once again, untreated and DMSO control groups did not show a significant difference.

Intrinsic differences in clonogenic capacity were evident among cell lines, with RT4 cells forming the largest and most compact colonies, followed by MDA-MB-231, whereas T98G cells formed smaller and fewer colonies. Both extracts decreased colony size across all cell lines, indicating impaired proliferative expansion of surviving cells. The extent of this effect varied between cell lines, reflecting differences in their inherent clonogenic potential. These findings demonstrate that both *L. stoechas* L. extracts reduce clonogenic survival across all tested cancer cell lines, with LsDE showing a stronger inhibitory effect than LsFE.

### 3.2. L. stoechas L. Extracts Increase PI-Positive Cell Population in MDA-MB-231 Cells

**Figure 2:**
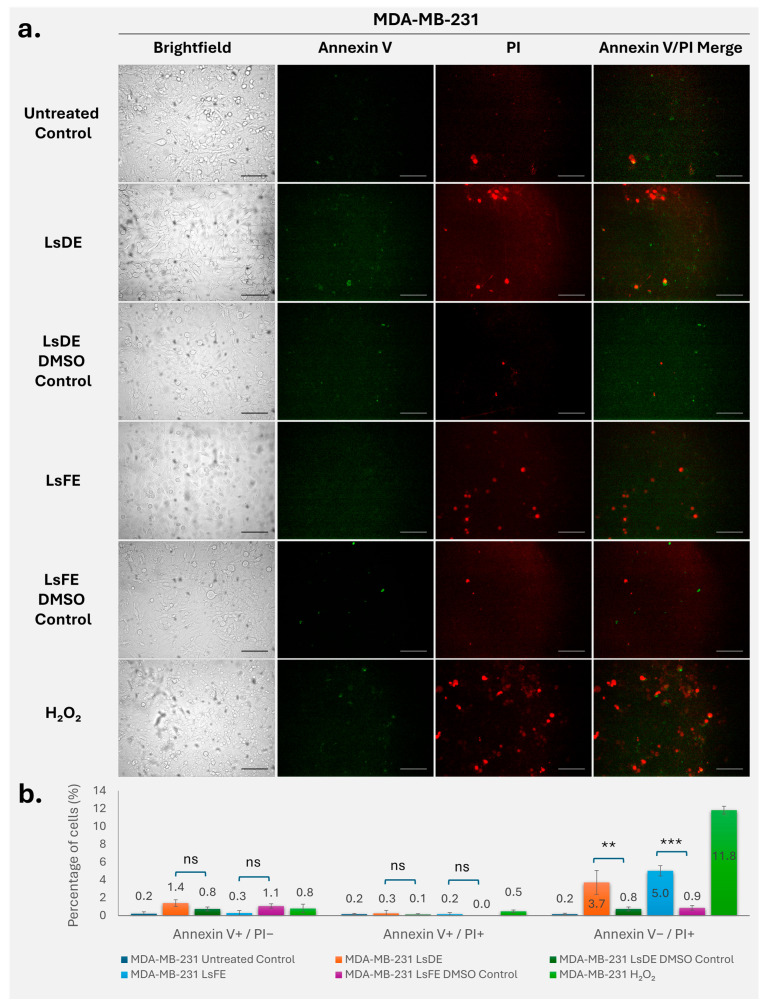
Annexin V–FITC/PI analysis of apoptosis in MDA-MB-231 cells following treatment with LsDE and LsFE. (**a**) Representative brightfield and fluorescence microscopy images of MDA-MB-231 cells following treatment with LsDE, LsFE, their respective DMSO vehicle controls, and H_2_O_2_ as a positive control for cell death (40× magnification). Annexin V–FITC staining (green) indicates phosphatidylserine externalization, a marker of early apoptosis, while PI (red) staining indicates loss of membrane integrity associated with late apoptosis or necrosis. Merged images show co-localization of Annexin V and PI signals, representing late apoptotic or secondary necrotic cells. Scale bars: 25 µm. (**b**) Quantitative analysis of apoptotic and necrotic cell populations expressed as percentages of total cells. Early apoptotic cells (Annexin V^+^/PI^−^), late apoptotic cells (Annexin V^+^/PI^+^), and necrotic cells (Annexin V^−^/PI^+^) were quantified. Data are presented as mean ± SD from three independent experiments. Statistical significance was determined by one-way ANOVA followed by Tukey’s post hoc test. ns, not significant; ** *p* < 0.01; *** *p* < 0.001 compared with untreated control.

Annexin V–FITC and PI staining were performed to evaluate apoptosis and membrane integrity changes in MDA-MB-231 cells after treatment with LsDE, LsFE, their respective DMSO vehicle controls, and H_2_O_2_ as a positive control (Figure 2a,b). Representative fluorescence images show low baseline Annexin V and PI staining in untreated and DMSO control groups, while extract-treated and H_2_O_2_-treated cells showed increased PI-positive staining (Figure 2a). Quantitative analysis showed that the percentage of early apoptotic cells (Annexin V^+^/PI^−^) was low in all groups and there was no significant difference between LsDE and its DMSO control, or between LsFE and its DMSO control (Figure 2b). Similarly, the late apoptotic group (Annexin V^+^/PI^+^) remained minimal across all conditions, and neither LsDE nor LsFE treatment resulted in a statistically significant change compared to their respective vehicle controls (Figure 2b). In contrast, analysis of the PI-positive/Annexin V-negative population (Annexin V^−^/PI^+^) showed a significant increase following treatment with LsDE and LsFE. LsDE treatment significantly increased the proportion of Annexin V^−^/PI^+^ cells (~3.7%) compared to its DMSO control (~0.8%) (*p* < 0.01). Similarly, LsFE treatment resulted in a significantly higher percentage of Annexin V^−^/PI^+^ cells (~5.0%) compared to its DMSO control (~0.9%) (*p* < 0.001). As expected, H_2_O_2_ treatment caused the highest proportion of PI-positive cells (~11.8%), confirming effective induction of cell death and validating the assay. These findings collectively show that *L. stoechas* L. extracts increase the PI-positive cell population in MDA-MB-231 cells compared to their respective vehicle controls, while Annexin V-defined early- and late-apoptotic fractions remain low and are not significantly affected under the tested conditions.

### 3.3. L. stoechas L. Extracts Induce Apoptotic and Membrane Integrity Loss in RT4 Cells

**Figure 3:**
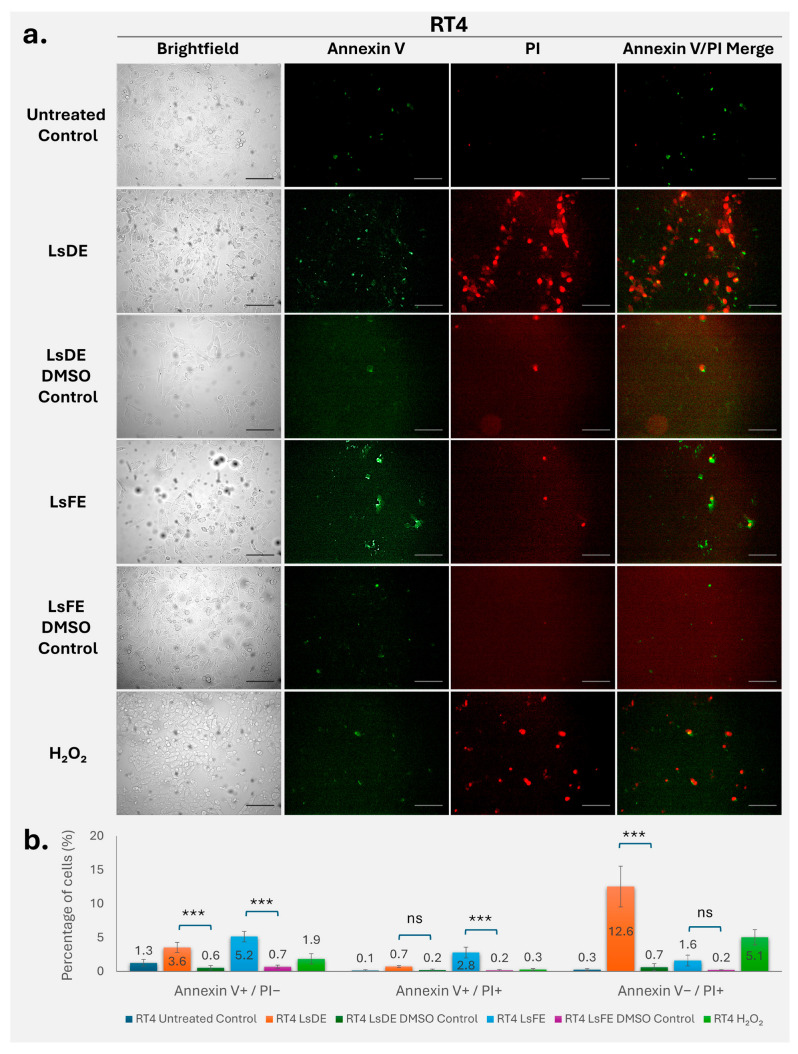
Annexin V–FITC/PI analysis of apoptosis in RT4 cells following treatment with LsDE and LsFE. (**a**) Representative brightfield and fluorescence microscopy images of RT4 bladder cancer cells treated with LsDE, LsFE, their corresponding DMSO vehicle controls, and H_2_O_2_ (40× magnification). Annexin V–FITC (green) indicates early apoptosis, whereas PI (red) indicates loss of membrane integrity. Scale bars: 25 µm. (**b**) Quantitative analysis of apoptotic and membrane-compromised cell populations. The percentages of early apoptotic (Annexin V^+^/PI^−^), late apoptotic (Annexin V^+^/PI^+^), and membrane-compromised (Annexin V^−^/PI^+^) cells were determined. Data represent mean ± SD (*n* = 3). Statistical comparisons were performed between each extract and its respective DMSO control using one-way ANOVA followed by Tukey’s post hoc test. ns, not significant; *** *p* < 0.001.

Annexin V–FITC and propidium iodide staining were used to evaluate apoptosis and membrane integrity in RT4 bladder cancer cells following treatment with *L. stoechas* L. extracts (Figure 3a,b). Untreated and vehicle control cells exhibited minimal Annexin V and PI staining, indicating low apoptosis-associated changes and confirming that DMSO alone did not significantly affect cell viability. Quantitative analysis revealed that LsDE significantly increased the proportion of early apoptotic cells (Annexin V^+^/PI^−^) to 3.6%, compared with 0.6% in the corresponding DMSO control (*p* < 0.001). Similarly, LsFE treatment significantly increased early apoptotic cells to 5.2%, compared with 0.7% in its vehicle control (*p* < 0.001). Analysis of late apoptotic cells (Annexin V^+^/PI^+^) demonstrated that LsFE significantly increased this population to 2.8%, compared with 0.2% in its DMSO control (*p* < 0.001). In contrast, LsDE treatment did not significantly alter the late apoptotic population compared with its vehicle control. Importantly, LsDE treatment caused a significant increase in membrane-compromised cells (Annexin V^−^/PI^+^), reaching 12.6%, compared to only 0.7% in the DMSO control (*p* < 0.001). This increase exceeded that seen with H_2_O_2_ treatment (5.1%), indicating that LsDE strongly promotes loss of membrane integrity in RT4 cells under the tested conditions. These findings demonstrate that *L. stoechas* L. extracts induce apoptosis and impair membrane integrity in RT4 cells, where LsFE encourages apoptotic progression and LsDE significantly raises the number of cells with damaged membranes.

### 3.4. L. stoechas L. Extracts Exert Limited Apoptotic Effects in T98G Glioblastoma Cells

**Figure 4:**
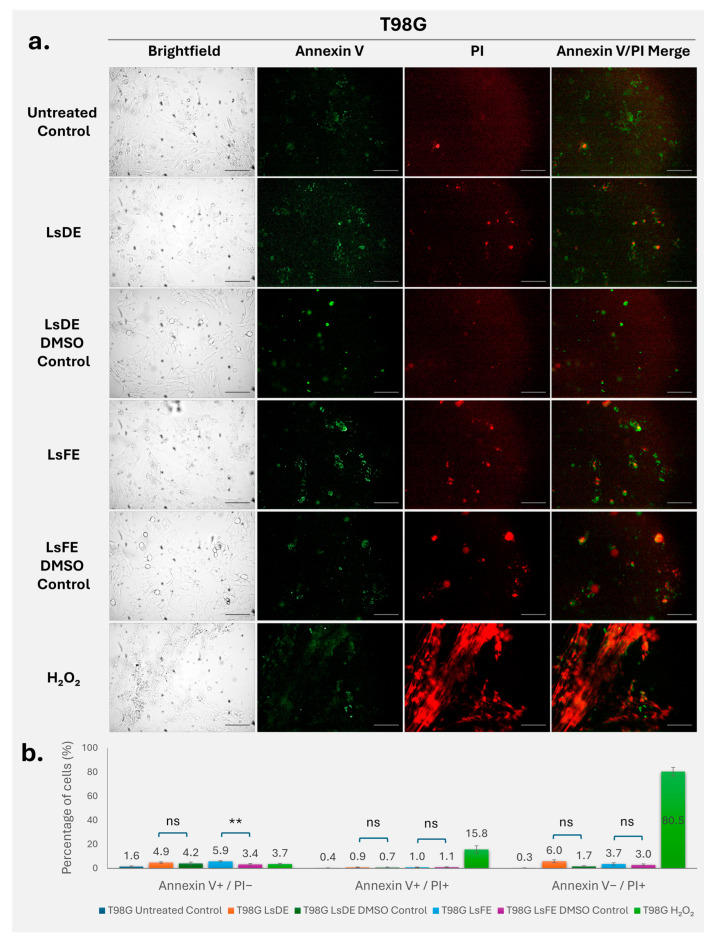
Annexin V–FITC/PI analysis of apoptosis in T98G cells following treatment with LsDE and LsFE. (**a**) Representative brightfield and fluorescence microscopy images of T98G glioblastoma cells treated with LsDE, LsFE, their respective DMSO vehicle controls, and H_2_O_2_ (40× magnification). Annexin V–FITC (green) indicates phosphatidylserine externalization associated with early apoptosis, whereas propidium iodide (PI, red) indicates loss of membrane integrity. Merged images show co-localization of Annexin V and PI signals. Scale bars: 25 µm. (**b**) Quantitative analysis of early apoptotic (Annexin V^+^/PI^−^), late apoptotic (Annexin V^+^/PI^+^), and membrane-compromised (Annexin V^−^/PI^+^) cell populations. Data represent mean ± SD (*n* = 3). Statistical comparisons were performed between each extract and its corresponding DMSO control using one-way ANOVA followed by Tukey’s post hoc test. ns, not significant; ** *p* < 0.01.

Annexin V–FITC and propidium iodide staining was used to evaluate apoptosis and membrane integrity changes in T98G glioblastoma cells following treatment with *L. stoechas* L. extracts (Figure 4a,b). The untreated and vehicle control groups showed low baseline levels of Annexin V and PI staining, which indicates minimal spontaneous apoptosis and confirms that DMSO alone does not significantly affect cell viability. Quantitative analysis showed that treatment with LsDE increased the proportion of early apoptotic cells (Annexin V^+^/PI^−^) to 4.9%, compared to 4.2% in the corresponding DMSO control; however, this difference was not statistically significant (Figure 4b, ns). In contrast, LsFE significantly raised the percentage of early apoptotic cells to 5.9%, compared with 3.4% in its vehicle control (*p* < 0.01). Analysis of late apoptotic cells (Annexin V^+^/PI^+^) revealed low overall levels across extract-treated and vehicle control groups, and neither LsDE nor LsFE treatment resulted in statistically significant differences compared with their respective DMSO controls. Similarly, analysis of membrane-compromised cells (Annexin V^−^/PI^+^) showed no statistically significant increase in the extract-treated cells compared with vehicle controls. In contrast, hydrogen peroxide treatment induced a marked increase in both late apoptotic cells (15.8%) and membrane-compromised cells (80.5%), confirming robust induction of cell death and validating the assay sensitivity. LsFE treatment significantly increased early apoptosis in T98G cells, whereas LsDE had no statistically significant effect. Neither LsDE nor LsFE significantly affected late apoptosis or membrane integrity compared with their respective vehicle controls.

### 3.5. L. stoechas L. Extracts Differentially Modulate BAX and BCL2 Expression and the BAX/BCL2 Ratio

**Figure 5:**
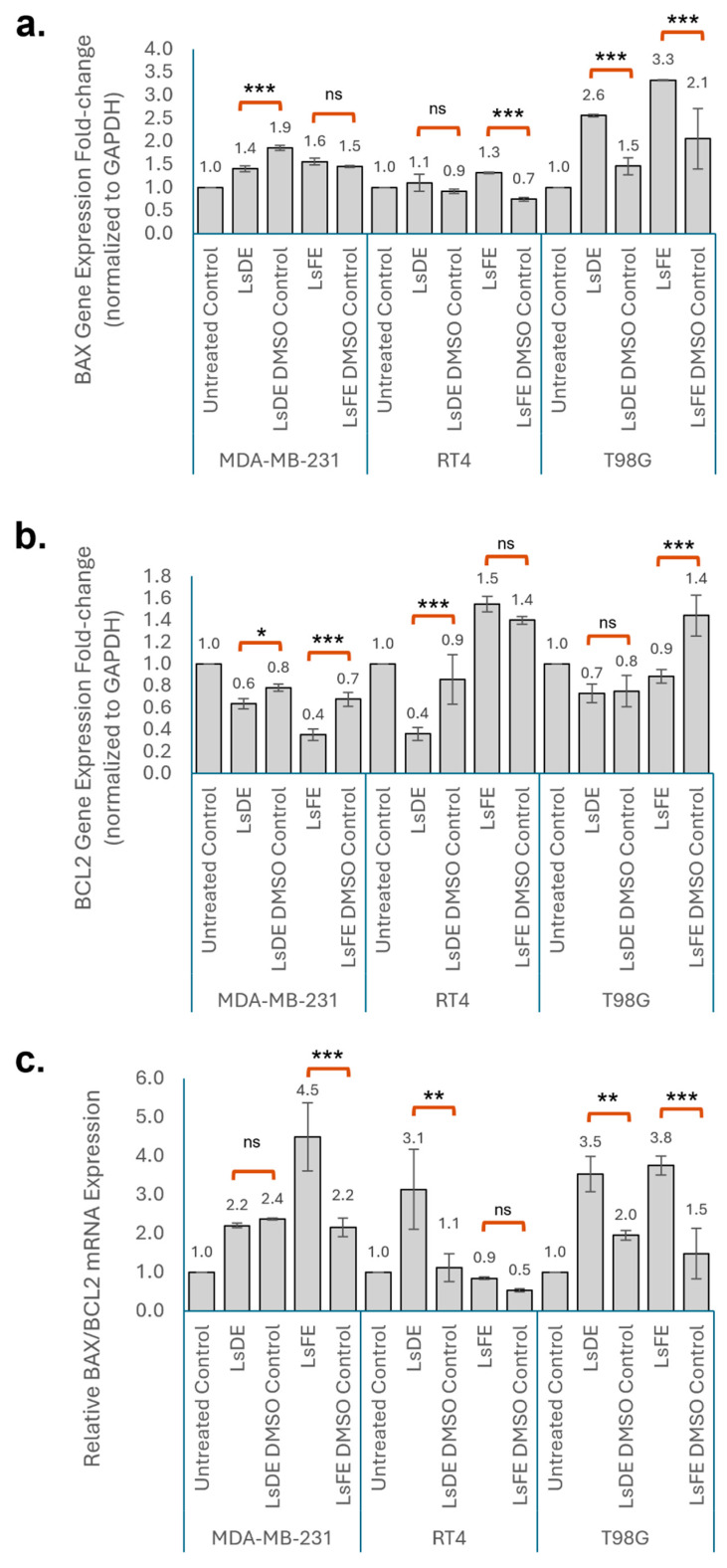
Effects of *L. stoechas* L. extracts on apoptosis-related gene expression in MDA-MB-231, RT4, and T98G cells. (**a**) Relative *BAX* mRNA expression levels normalized to *GAPDH*. (**b**) Relative *BCL2* mRNA expression levels normalized to *GAPDH*. (**c**) Relative *BAX/BCL2* mRNA expression ratio calculated from normalized expression values. Cells were treated with LsDE or LsFE and compared with their corresponding DMSO vehicle controls. Untreated cells served as baseline controls. Gene expression levels were determined by quantitative real-time PCR and are presented as fold change relative to untreated control. Data are shown as mean ± SD of independent experiments. Statistical significance was determined by one-way ANOVA followed by appropriate post hoc comparisons between extract-treated groups and their corresponding DMSO controls. ns, not significant; * *p* < 0.05; ** *p* < 0.01; and *** *p* < 0.001.

*BAX* mRNA expression was assessed following treatment with LsDE and LsFE in MDA-MB-231, RT4, and T98G cells (Figure 5a). In MDA-MB-231 cells, LsFE treatment increased BAX expression compared with its corresponding DMSO control, although this change did not reach statistical significance. Interestingly, LsDE treatment led to a significant reduction in *BAX* expression relative to its vehicle control. In RT4 cells, LsDE did not significantly change *BAX* expression relative to its DMSO control (1.1 vs. 0.9), while LsFE significantly increased *BAX* expression compared with its corresponding DMSO control (1.3 vs. 0.7). In T98G cells, both LsDE and LsFE significantly increased BAX expression relative to their vehicle controls (LsDE: 2.6 vs. 1.5; LsFE: 3.3 vs. 2.1).

*BCL2* mRNA expression (normalized to GAPDH) was evaluated following treatment with LsDE and LsFE in MDA-MB-231, RT4, and T98G cells (Figure 5b). In MDA-MB-231 cells, LsDE significantly decreased *BCL2* expression compared with its corresponding DMSO control (0.6 vs. 0.8), and LsFE also significantly reduced *BCL2* relative to its vehicle control (0.4 vs. 0.7). In RT4 cells, LsDE significantly reduced *BCL2* expression compared with the LsDE DMSO control (0.4 vs. 0.9), whereas LsFE did not produce a statistically significant change relative to its corresponding vehicle control (1.5 vs. 1.4; ns). In T98G cells, LsDE did not significantly alter *BCL2* expression compared with its DMSO control (0.7 vs.0.8; ns), while LsFE significantly increased *BCL2* expression relative to its vehicle control (0.9 vs. 1.4).

The relative *BAX/BCL2* mRNA expression ratio was evaluated following treatment with LsDE and LsFE in MDA-MB-231, RT4, and T98G cells (Figure 5c). In MDA-MB-231 cells, LsFE significantly increased the *BAX/BCL2* ratio compared with its corresponding DMSO control (4.5 vs. 2.2), whereas LsDE did not significantly change the ratio relative to its vehicle control (2.2 vs. 2.4; ns). In RT4 cells, LsDE significantly increased the *BAX/BCL2* ratio compared with the LsDE DMSO control (3.1 vs. 1.1), whereas LsFE did not produce a statistically significant change relative to its vehicle control. In T98G cells, both LsDE and LsFE significantly increased the *BAX/BCL2* ratio compared with their respective DMSO controls (LsDE: 3.5 vs. 2.0; LsFE: 3.8 vs. 1.5).

Collectively, analysis of *BAX*, *BCL2*, and the *BAX/BCL2* ratio indicates that *L. stoechas* L. extracts are associated with changes in apoptosis-related gene expression in a cell line-dependent manner. LsFE markedly increased the *BAX/BCL2* ratio in MDA-MB-231 and T98G cells, whereas LsDE significantly elevated the ratio in RT4 and T98G cells. These findings indicate that both extracts promote a pro-apoptotic transcriptional shift, although the magnitude and direction of changes in individual *BAX* and *BCL2* vary across cell lines.

### 3.6. Cell-Type-Specific Regulation of Bax/Bcl-2 Axis and PARP Cleavage by L. stoechas L. Extracts

**Figure 6:**
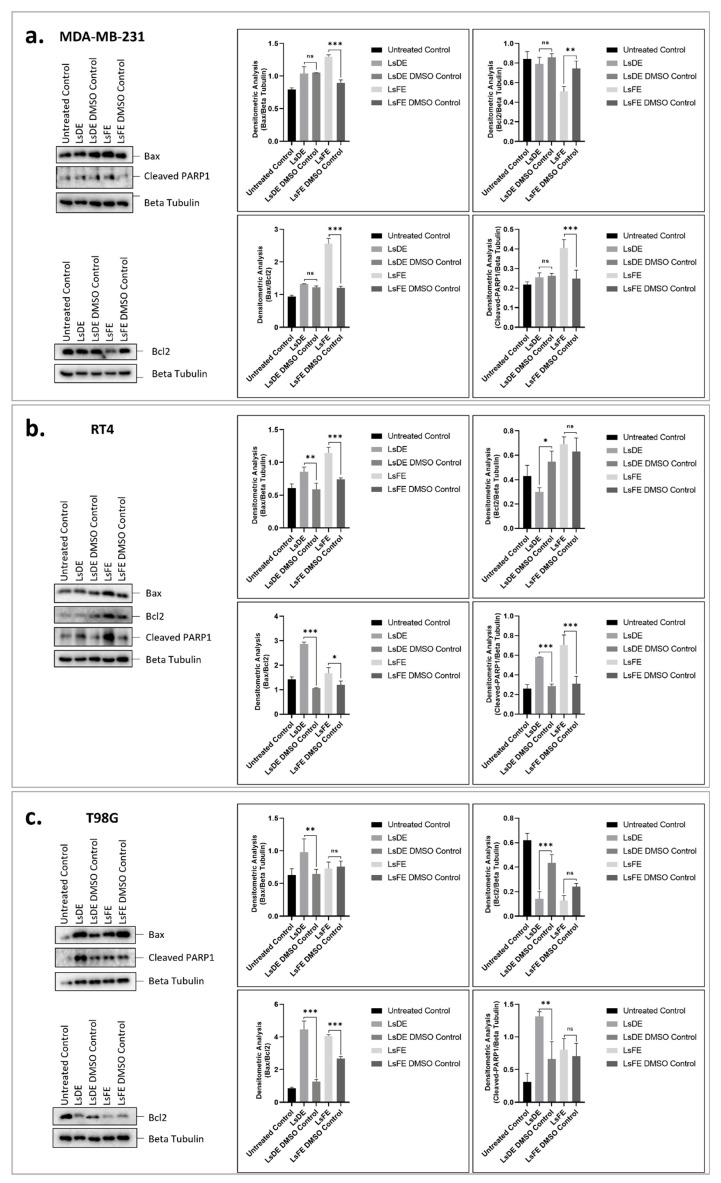
Effects of LsDE and LsFE on apoptotic protein expression in cancer cell lines. Western blot analysis showing the effects of LsDE and LsFE treatments on apoptotic markers in (**a**) MDA-MB-231, (**b**) RT4, and (**c**) T98G cells. Protein levels of Bax, Bcl-2, and cleaved PARP1 were analyzed following treatment with LsDE and LsFE, along with their respective DMSO controls. β-Tubulin was used as the loading control. Densitometric analyses of Bax/β-tubulin, Bcl-2/β-tubulin, cleaved PARP1/β-tubulin, and Bax/Bcl-2 ratios are presented in the corresponding bar graphs. Data are expressed as mean ± SD from three independent experiments. Statistical significance was determined by one-way ANOVA followed by appropriate post hoc analysis. Comparisons were primarily performed between each treatment group and its corresponding DMSO control. ns, not significant; * *p* < 0.05; ** *p* < 0.01; and *** *p* < 0.001. Representative brightfield and chemiluminescent images of the Western blot membranes are shown in Supplementary Figure S1.

The effects of LsDE and LsFE on apoptotic signaling were comparatively evaluated in MDA-MB-231, RT4, and T98G cell lines by Western blot analysis. Protein levels of Bax, Bcl-2, and cleaved PARP1 were normalized to β-tubulin, and apoptotic balance was further assessed by calculating the Bax/Bcl-2 ratio (Figure 6a–c).

In MDA-MB-231 cells (Figure 6a), LsDE did not significantly alter Bax expression, whereas LsFE markedly increased Bax levels (1.295 vs. 0.895; 1.45-fold; *p* < 0.001). Consistently, Bcl-2 expression remained unchanged in the LsDE group, while LsFE significantly suppressed Bcl-2 levels (0.510 vs. 0.744; *p* < 0.01). The Bax/Bcl-2 ratio significantly increased following LsFE treatment (2.555 vs. 1.208; 2.12-fold; *p* < 0.001), whereas LsDE did not cause a significant change. Cleaved PARP1 levels paralleled these findings, showing a significant increase exclusively in the LsFE-treated group (0.404 vs. 0.248; 1.63-fold; *p* < 0.001). These results indicate that LsFE treatment is associated with changes in apoptosis-related protein expression in MDA-MB-231 cells.

In RT4 cells (Figure 6b), both extracts significantly altered the expression of apoptosis-related proteins. Bax expression was significantly increased following both LsDE (0.858 vs. 0.589; 1.46-fold; *p* < 0.01) and LsFE treatment (1.145 vs. 0.745; 1.54-fold; *p* < 0.001). However, Bcl-2 suppression was significant only in the LsDE group (0.300 vs. 0.549; ~45% reduction; *p* < 0.05), whereas LsFE did not significantly affect Bcl-2 levels. Consequently, the Bax/Bcl-2 ratio increased more robustly in the LsDE group (2.865 vs. 1.072; 2.67-fold; *p* < 0.001) compared to LsFE (1.675 vs. 1.206; 1.39-fold; *p* < 0.05). Both treatments significantly elevated cleaved PARP1 levels (LsDE: 2.04-fold; LsFE: 2.26-fold; *p* < 0.001), indicating increased apoptotic signaling. These results indicate that although both extracts were associated with changes in apoptosis-related protein levels in RT4 cells, LsDE has a stronger effect on the Bax/Bcl-2 ratio.

In T98G glioblastoma cells (Figure 6c), changes in apoptosis-related markers were more evident with LsDE treatment. Bax expression significantly increased in the LsDE group (0.981 vs. 0.646; 1.52-fold; *p* < 0.01), while LsFE produced no significant change. LsDE significantly suppressed Bcl-2 expression (0.142 vs. 0.436; ~67% reduction; *p* < 0.001), accompanied by an increase in the Bax/Bcl-2 ratio (4.446 vs. 1.273; 3.49-fold increase; *p* < 0.001). Although LsFE also increased the Bax/Bcl-2 ratio (4.053 vs. 2.677; 1.51-fold increase; *p* < 0.001), it did not significantly modulate Bax or Bcl-2 individually. Cleaved PARP1 levels were significantly elevated only in the LsDE group (1.79-fold; *p* < 0.01). These findings suggest that LsDE may exert a stronger effect on mitochondrial apoptotic signaling in T98G cells.

Comparative analysis across all three cell lines revealed different response patterns. LsFE produced greater changes in apoptosis-related markers in MDA-MB-231 breast cancer cells, whereas LsDE showed stronger effects in T98G glioblastoma cells. In RT4 cells, both extracts increased apoptotic signaling, with LsDE showing a greater change in the Bax/Bcl-2 ratio.

When the findings from clonogenic survival, Annexin V/PI staining, gene expression analysis, and Western blotting are considered together, a cell type–dependent response pattern was observed. MDA-MB-231 cells showed the most remarkable changes in apoptosis-related markers following LsFE treatment, characterized by an elevated Bax/Bcl-2 ratio, and enhanced PARP1 cleavage. RT4 cells responded to both extracts, showing decreased clonogenic survival together with modulation of apoptosis-related markers, although LsDE produced a more substantial shift in mitochondrial apoptotic balance. In contrast, T98G glioblastoma cells exhibited comparatively limited evidence of apoptosis at the cellular level despite detectable modulation of apoptosis-related gene and protein expression, with LsDE producing the most evident pro-apoptotic effects in this model. Overall, these results indicate that apoptotic responses to *L. stoechas* L. extracts differ among tumor cell types, with LsFE more effective in MDA-MB-231 cells, whereas LsDE shows a greater effect in T98G cells, while RT4 cells respond to both extracts.

## 4. Discussion

The present study aimed to explore potential mechanisms underlying the anticancer effects of *L. stoechas* L. ethanol extracts by integrating long-term survival assays, phenotypic apoptosis analysis, targeted gene expression analysis, and protein-level validation across different cancer cell lines. While the anticancer potential of *L. stoechas* has been previously reported, existing studies are largely limited to cytotoxicity or selected apoptosis-related endpoints and have predominantly focused on essential oils rather than ethanol-based extracts. In addition, comparative evaluation across different types of cancer remains limited. In this context, the present study provides a more integrated assessment of extract-dependent and cell line-dependent responses. The findings indicate that both LsDE and LsFE impair proliferative capacity and modulate markers related to apoptotic signaling in a cancer type-dependent manner.

Clonogenic survival assays are widely used to assess the long-term proliferative capacity of cancer cells following therapeutic intervention, as they reflect the ability of single cells to sustain proliferative growth over time [21]. Consistent with previous studies showing that natural product–derived extracts can suppress clonogenic survival in different cancer models [22,23], both LsDE and LsFE reduced colony formation in all tested cell types. This finding indicates that *L. stoechas* L. extracts impair sustained proliferative potential beyond short-term cytotoxic effects. The more pronounced reduction observed with LsDE in certain models suggests that differences in extract composition may contribute to long-term growth suppression, as variability in phytochemical content is known to influence biological activity [24]. Additionally, intrinsic differences in baseline clonogenic capacity among cell lines, exemplified by the larger and more compact colonies observed in RT4 cells, may further contribute to variability in treatment response [21]. These findings suggest that *L. stoechas* L. extracts reduce long-term cancer cell survival in an extract- and cell type–dependent manner, warranting further investigation into the molecular mechanisms underlying these growth-inhibitory effects.

To determine whether the reduction in long-term proliferative capacity was associated with activation of cell death pathways, Annexin V/PI staining was performed to evaluate apoptotic and membrane integrity changes. *L. stoechas* L. extracts induced, cell line–dependent patterns of Annexin V and PI staining across MDA-MB-231, RT4, and T98G cells. Increased PI-positive populations in MDA-MB-231 cells suggest membrane integrity loss, which may occur independently of classical early apoptotic progression or reflect rapid transition through apoptotic stages [25]. Similar patterns have been reported in breast cancer models treated with phytochemical-rich extracts, where membrane permeability changes were observed alongside limited early apoptotic detection [26]. In RT4 cells, the increase in Annexin V^+^ populations is consistent with apoptotic involvement, whereas T98G cells exhibited comparatively limited responses, in line with their known resistance to apoptosis [27,28]. These findings indicate that *L. stoechas* L. extracts modulate cell death-related responses in a tumor-type–dependent manner with membrane-associated effects more prominent in MDA-MB-231 cells, and combined apoptotic features in RT4 cells. T98G cells displayed a comparatively modest response under the tested conditions. Annexin V/PI analysis in this study was performed using fluorescence microscopy, which allows assessment of staining patterns and membrane integrity. While flow cytometry may provide more detailed quantification of apoptotic populations, the present approach was supported by analysis of multiple fields and independent experiments.

To clarify whether these phenotypic changes were accompanied by transcriptional modulation of intrinsic apoptotic regulators, *BAX* and *BCL2* gene expressions were analyzed by qRT-PCR. The findings indicate cell type-dependent modulation of apoptosis-related gene expression following LsDE and LsFE treatments. In particular, changes in the *BAX/BCL2* ratio across different cell types reflect shifts in the balance between pro- and anti-apoptotic signaling. Alterations in *BCL2* family gene expression are frequently associated with responsiveness to anticancer agents, targeting mitochondrial apoptotic pathways [29,30]. The extract-specific differences observed across cell lines may reflect inherent molecular heterogeneity, since baseline expression levels and regulatory control of *BAX* and *BCL2* vary between tumor types [31]. Interestingly, previous work investigating the anticancer effect of *L. stoechas* L. flower ethanolic extract in colorectal cancer cells demonstrated induction of apoptosis through *TP53* and *CASP3* upregulation despite downregulation of *BAX* expression, suggesting that *L. stoechas*–mediated apoptotic signaling may, in certain contexts, proceed independently of classical *BAX*-driven mechanisms [32]. These findings further suggest that the apoptotic responses to *L. stoechas* L. extracts may vary depending on tumor type and molecular background. Overall, these findings suggest that *L. stoechas* L. extracts influence apoptotic gene regulation primarily through modulation of the *BAX/BCL2* axis, with the direction and magnitude of response depending on cellular context.

To assess whether transcriptional alterations were reflected at protein-level, Western blot analysis was performed to evaluate Bax, Bcl-2, and cleaved PARP1 expression. The results demonstrated tumor type–dependent modulation of apoptosis-related protein levels following LsDE and LsFE treatments. The coordinated changes observed in Bax, Bcl-2 and cleaved PARP1 are consistent with modulation of apoptosis-related signaling processes [29].

In MDA-MB-231 cells, LsFE showed the strongest pro-apoptotic pattern, including reduced Bcl-2 levels, an increased Bax/Bcl-2 ratio, and elevated cleaved PARP1. PARP1 cleavage is widely recognized as a marker of caspase-dependent apoptosis [33], consistent with downstream apoptotic processes in this model. In contrast, LsDE produced comparatively moderate effects in MDA-MB-231 cells, indicating differential sensitivity to the extract. In RT4 cells, both extracts modulated the expression of apoptosis-related proteins, with LsDE showing a stronger effect on the Bax/Bcl-2 ratio. In T98G cells, LsDE demonstrated the most robust changes in apoptosis-related markers by elevation of the Bax/Bcl-2 ratio, accompanied by increased PARP1 cleavage. These findings indicate that LsDE and LsFE are associated with changes in expression levels of apoptotic proteins by regulating the Bax/Bcl-2 axis and downstream PARP1 cleavage, with the dominant extract varying by tumor type. Changes in Bax/Bcl-2 ratio and PARP1 cleavage are consistent with apoptotic signaling; however, future studies incorporating additional apoptotic markers will further refine pathway-level interpretation.

While the present study focused on mechanistic evaluation in cancer cell lines, previous work [14] has demonstrated comparatively lower sensitivity of non-cancerous cells to *L. stoechas* L. ethanol extracts, supporting a degree of selectivity consistent with the differential responses observed in this study. When considered together, the clonogenic, phenotypic, transcriptional, and protein-level findings converge to indicate that *L. stoechas* L. extracts appear to modulate components of the apoptotic pathway. However, the dominant extract and the magnitude of the response vary by tumor type, reflecting differences in apoptotic threshold and molecular contexts. Differences observed between LsDE and LsFE may reflect preparation-related variations that could influence their biological effects. This tumor-dependent responsiveness highlights the biological complexity of extract-based interventions and suggests that phytochemical composition may influence pathway selectivity [26]. Phytochemical characterization of *L. stoechas* L. ethanol extracts in our previous study revealed diverse bioactive compounds, including phenolic acids, coumarin derivatives, terpenoid-related molecules, and oxidized fatty acids. Certain constituents, such as tyrosol and coumarin-type metabolites, have been reported to be associated with the regulation of apoptosis and cancer-related signaling pathways [34,35]. Although the present study does not directly investigate the contribution of individual compounds, the observed modulation of apoptotic markers may be associated with the combined effects of these bioactive constituents.

Although *L. stoechas* L. has attracted increasing attention for its biological activities, the majority of available studies have predominantly focused on essential oil composition, antioxidant capacity, or short-term cytotoxicity assays. Mechanistic investigations exploring apoptotic signaling are comparatively limited, and detailed characterization of mitochondrial pathway engagement at both transcriptional and protein levels remains insufficiently addressed, particularly for ethanol-based extracts. Moreover, direct comparative evaluation across distinct tumor types is largely lacking. In this context, the present study contributes to the field by providing an integrated analysis of long-term proliferative suppression together with phenotypic, transcriptional, and protein-level assessments of apoptotic regulation in multiple cancer models, thereby extending the current understanding of *L. stoechas*–mediated anticancer mechanisms.

## 5. Conclusions

This study demonstrates that ethanol extracts of *L. stoechas* L. impair long-term proliferative capacity and are associated with changes in apoptosis-related signaling in a tumor type-dependent manner. LsFE shows stronger apoptotic effects in MDA-MB-231 breast cancer cells, whereas LsDE shows greater effects in T98G glioblastoma cells, while RT4 cells respond to both extracts through partially distinct patterns of apoptotic regulation. Changes in the Bax/Bcl-2 balance together with PARP1 cleavage are consistent with involvement of mitochondrial apoptotic mechanisms. These findings suggest that *L. stoechas* L. extracts may differentially modulate apoptotic pathways depending on tumor context and merit further investigation in anticancer research.

## Data Availability

The original contributions presented in this study are included in the article/Supplementary Materials. Further inquiries can be directed to the corresponding author.

## Supplementary Materials

The following supporting information can be downloaded at https://www.mdpi.com/article/10.3390/pharmaceutics18040500/s1, Supplementary Figure S1. Brightfield and chemiluminescent images of Western blot membranes in MDA-MB-231, RT4, and T98G cells.
