# Supplementary figures and images for: Lavandula stoechas Ethanol Extracts Induce Apoptosis in Breast, Bladder, and Glioblastoma Cancer Cells
Source: Pharmaceutics. 2026 Apr 18;18(4):500. doi: 10.3390/pharmaceutics18040500 (PMC13118909; doi:10.3390/pharmaceutics18040500)

Supplementary Figure S1

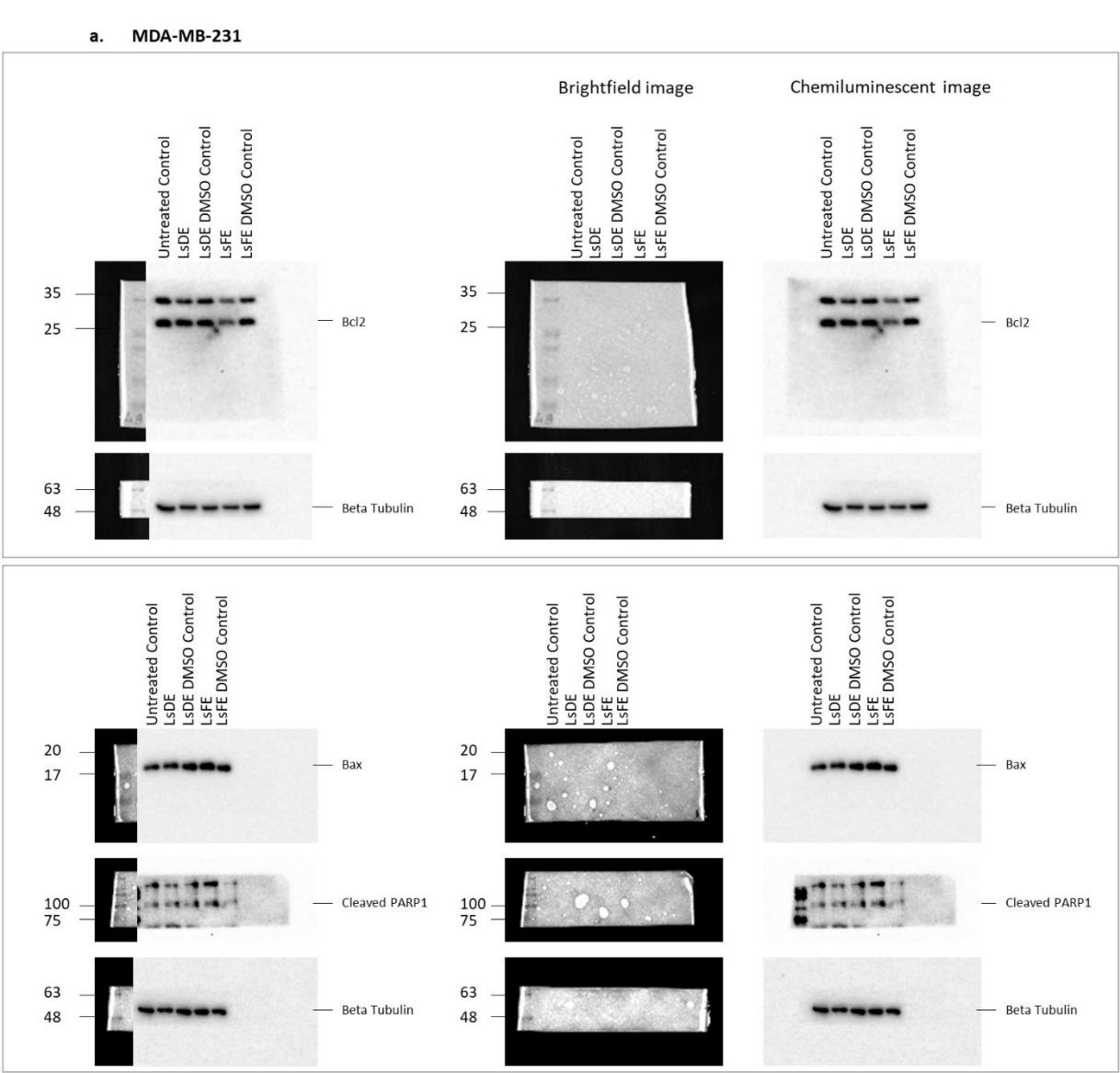

**b. RT4**

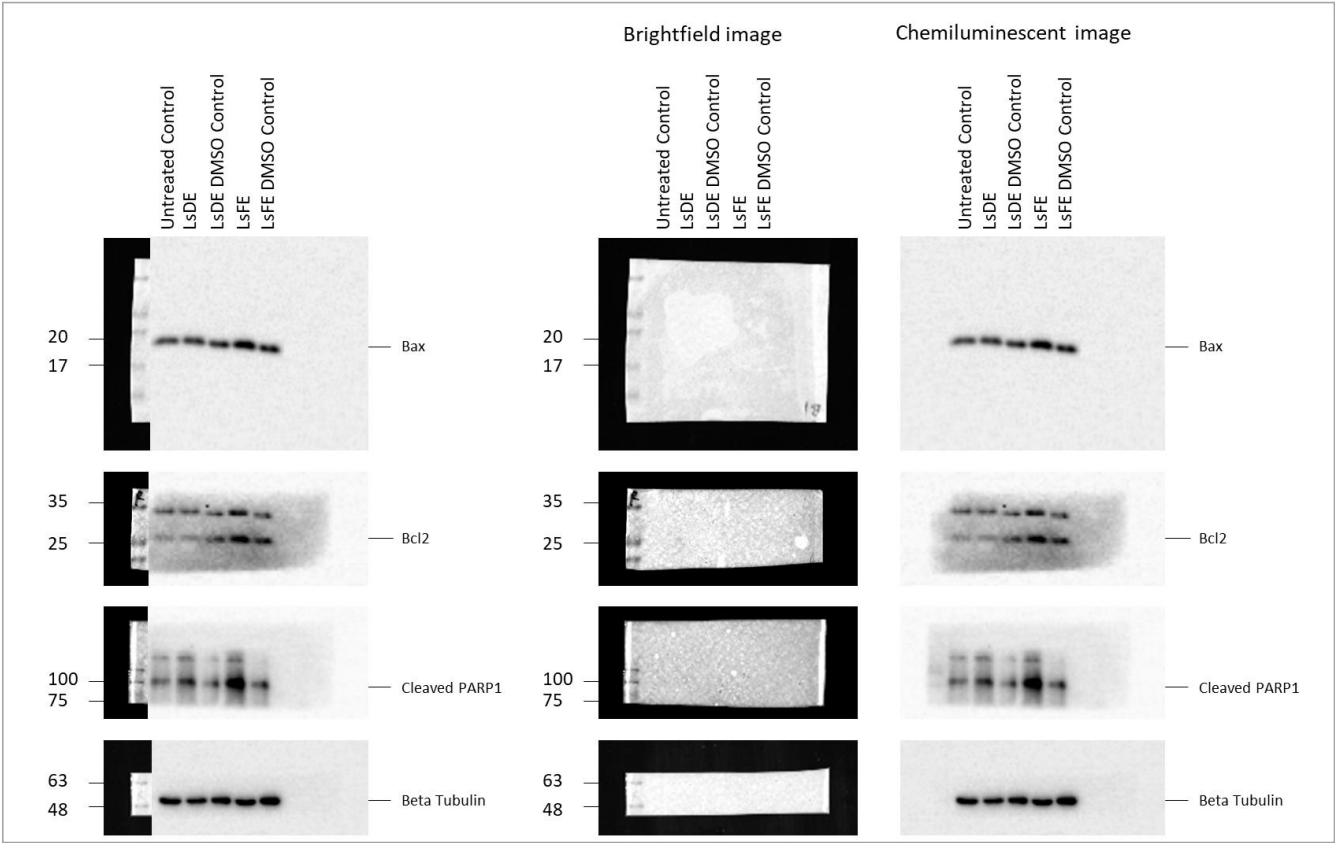

c. T98G

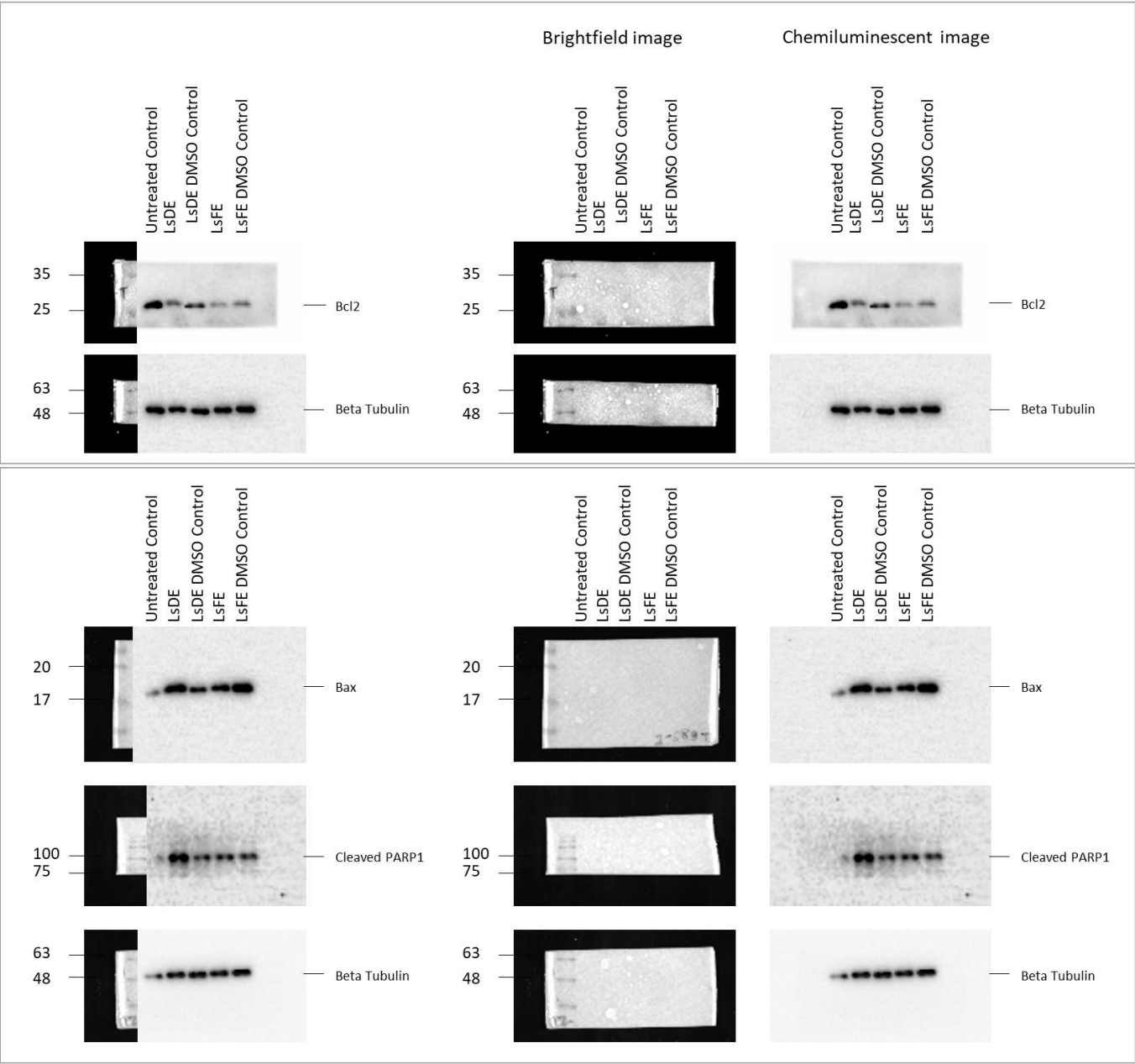

Supplement: Supplementary file 1 [file pharmaceutics-18-00500-s001.zip › pharmaceutics-4211537-supplementary.pdf]
